# Supplementary material for: Midbrain projection to the basolateral amygdala encodes anxiety-like but not depression-like behaviors
Source: Nat Commun. 2022 Mar 22;13:1532. doi: 10.1038/s41467-022-29155-1 (PMC8940900; doi:10.1038/s41467-022-29155-1)
Supplement: Supplementary file 1 — Supplementary information [file 41467_2022_29155_MOESM1_ESM.pdf]

Supplementary Information:

Figure Legends:

Supplementary Fig. 1. CSDS induces anxiety-like behaviors independently of social avoidance behaviors. a-g Supplementary results of C57BL6/J mouse behaviors depicted in main Fig. 1. **a** Time (%) in EPM open arms of CSDS-exposed mice (CSDS n=66 mice: n=39 AD + n=27 A mice) compared to stress-naïve CTL mice (n=28 control) (Mann-Whitney test: U=361 p=0.001). **b** Time (s) in EPM open arms, closed arms and center in n=28 control, n=39 AD and n=27 A mice (Kruskal-Wallis test, open: $H_{2/91}$=24.61 p=0.001; Z=4.887 p=0.001; Z=3.336 p=0.003; Z=1.241 p=0.64, closed: $H_{2/91}$=20.44, p=0.001; Z=1.293 p=0.59; Z=2.913 p=0.01; Z=4.479 p=0.001, center: $H_{2/91}$=1.36, p=0.51), and related locomotion and velocity during the EPM test (Kruskal-Wallis test, locomotion: $H_{2/91}$=7.41 p=0.051, velocity: $H_{2/91}$=0.58, p=0.74). **c** Spearman correlation analyses show that the time (%) in open arms does not correlate with the social interaction behaviors of the stress-naïve CTL mice (r=0.079, p=0.98, n=28). **d** Time (%) in OFT center of CSDS-exposed mice (CSDS n=40 mice: n=19 AD and n=21 A mice) compared to stress-naïve CTL mice (n=15 control; Mann-Whitney test: U=92.5, p=0.001). **e** Social interaction behaviors of mice depicted in main figure 1i-l (Paired t-tests: t=2.84 p=0.008; t=5.06 p=6.9e-05; t=4.09 p=0.0002), blue circles represent mean ± s.e.m., and respective social interaction ratio in CTL, AD and A mice (ANOVA, $F_{2/91}$=36.02 p=1.54e-10; t=6.758 p=1.2e-08, t=0.35 p=0.99; t=7.75 p=3.3e-10 n=15-21 mice). **f** The time (s) in open field center revealed that both AD and A mice have a decreased time spent in open field center compared to stress-naïve CTL mice (Kruskal-Wallis test, $H_{2/52}$=17.86, p=3.17e-06, Z=2.64 p=0.02; Z=4.127 p=0.001; Z=1.62 p=0.31, for n=15 CTL, n=19 AD and n=21 A mice), and velocity and locomotion during the OFT show no differences between CTL, AD and A mice (ANOVA, velocity: $F_{2/52}$=0.31 p=0.4, locomotion: $F_{2/52}$=0.94 p=0.32). **g** Spearman correlation analyses show that the time (%) in open field center does not correlate with the social interaction behaviors of CTL mice (r=0.16, p=0.56, n=15). **h** Experimental timeline for a separate cohort of mice. **i** Spearman correlation analyses of the social interaction behavior before (day -7) and after CSDS (day 11) and respective direct comparison analyses in AD (Top right) and A mice (Bottom right; respective

analyses: r=-0.023, p=0.91, n=29; AD mice: paired t-test, t=5.87 p=0.0001, n=19; A mice: paired t-test, t=0.76 p=0.46, n=10). **j** Spearman correlation analyses of the time in open arms before (day -4) and after CSDS (day 14, r=0.445 p=0.016, n=19) and respective direct comparison analyses in AD (Top right, t=5.87 p=0.0001) and A mice (Bottom right; t=2.28, p=0.04, n=10). **k** Spearman correlation analyses showing that the social interaction behavior after CSDS does not correlate with the social interaction behaviors before CSDS in control mice (r=-0.601; t=0.77, p=0.47, n=7). **l** Spearman correlation analyses showing that the time (%) in open arms after CSDS does not correlate with the time (%) in open arms before CSDS in CTL mice (r=0.071; t=2.07, p=0.09, n=7). In all panels, data are represented as mean ± s.e.m. for n number of mice; two-sided statistical analyses and post hoc corrected tests were performed, *p<0.05, **p<0.01, ***p<0.001.

Supplementary Fig. 2. CSDS induces anxiety-like behaviors independently of reduced preference for natural rewards. a Experimental timeline for a separate cohort of C57BL6/J mice composed of n=23 CTL, n=25 AD and n=21 A mice, **b** Social interaction ratio (ANOVA, $F_{2/66}$=17.39 p<0.0001; t=3.42 p=0.003; t=5.85 p<0.0001; t=2.26 p=0.39), preference for female urine sent over water sent (ANOVA, $F_{2/66}$=4.615 p=0.0057; t=2.97 p=0.012; t=2.75 p=0.015; t=0.14 p=0.887), sucrose preference (ANOVA, $F_{2/66}$=5.594 p=0.014; t=2.01 p=0.048; t=2.921 p=0.009; t=0.95 p=0.34), percentage of time spent in EPM open arms (ANOVA, $F_{2/66}$=6.830 p=0.002; t=3.34 p=0.004; t=3.04 p=0.01; t=0.16 p=0.99), and percentage of time spent in OFT center (ANOVA, $F_{2/66}$=6.607 p=0.002; t=2.6 p=0.034; t=3.49 p=0.002; t=1.02 p=0.93). Bars represent mean ± s.e.m. **c** Spearman correlation analyses showing that the preference for female urine and sucrose preference correlate with the social interaction behaviors in AD and A mice (r=0.55 p<0.001, r=0.34, p=0.02, n=46), but not with percentage of time spent in OFT center and percentage of time spent in EPM open arms (r=-0.06 p=0.65, r=-0.07 p=0.64, n=46). **d** Spearman correlation analyses showing that preference for female urine and sucrose preference do not correlate with the time (%) in EPM open arms in AD and A mice (r=-0.11 p=0.44, r=-0.12, p=0.40, n=46) while percentage of time spent in OFT center correlates with the time (%) in EPM open arms (r=0.39, p=0.006, n=46). **e** Same as c in stress naïve CTL mice (r=0.63, p=0.001, r=0.49, p=0.016, r=-0.01 p=0.95, r=0.19, p=0.36, n=23). **f** Same as d in

stress naïve CTL mice (r=0.19 p=0.36, r=-0.04, p=0.82, r=0.43 p=0.04, n=23). In all panels, data are represented as mean ± s.e.m.; two-sided statistical analyses and post hoc corrected tests were performed, *p<0.05, **p<0.01, ***p<0.001.

Supplementary Fig. 3. Circuit-probing and electrophysiological strategies to probe firing rate of retrobead-labeled VTA→BLA putative dopamine neurons and virally labeled VTA→BLA dopamine neurons in CTL, AD and A mice. a Experimental strategy used to label the potent co-projection of VTA neuron to both NAc and BLA, two viral vectors were bilaterally injected: AAVrg-tdTomato vectors in the NAc and AAVrg-eGFP vectors in the BLA of the same mice (scale bar=500 and 100 μm) in 5 C57BL/6J mice. **b** (Left) Confocal images showing expression of tdTomato in VTA→NAc neurons and eGFP in VTA→BLA neurons; (Center) quantification showing that amongst 2100 labeled neurons only 2.7% neurons were labeled for both eGFP and tdTomato and (Right) number of labeled VTA projecting neurons per projection. This experiment was performed in a VTA circuit-specific manner (empty circle, 5 mice) and in a VTA dopamine cell- and circuit-specific manner using Cre-dependent vectors in 4 TH-BAC-Cre mice (see methods section). **c** Experimental timeline. **d** Schematics of the two retrograde-targeting methods for circuit- and/or cell-specific *ex vivo* electrophysiological recording: (Top) C57bl/6J mice injected with green luma retrobeads and in (Bottom) Th-BAC-Cre mice injected with retrograding AAVrg-DIO-eYFP, and related confocal images showing co-expression of (Top) Th labelling (red) with Luma retrobeads (green) and (Bottom) Th labelling (red) with AAVrg-DIO-eYFP expression (green) in TH-BAC-Cre mice (scale bar, 100 μm), quantification shows 94% collocalization (2-3 sections per mouse from 4 mice). **e-g** Firing activity is similar in retrobead-labeled VTA→BLA putative dopamine neurons in C57BL6/J mice and virally labeled VTA→BLA dopamine neurons in TH-BAC-Cre mice. **e** Sample traces of VTA→BLA neuron *ex vivo* cell-attached recordings in control mice (scale bar=0.2mV), the average firing rate is not statistically different (t.test, t=0.24, p=0.81, n=16,15). **f** Same as **e** in AD mice (t.test, t=0.23, p=0.81, n=14,32). **g** Same as **e** in A mice (t.test, t=1.49, p=0.14, n=9,21). Bars represent mean ± s.e.m. for n=number of neurons. **h** Spearman correlation analyses of VTA→BLA dopamine neuron firing with the

social interaction behavior and with time (%) in EPM open arms in stress-naïve CTL mice (n=7, p=0.35, p=0.048, 3-7 neurons per mouse, combined C57BL6/J and TH-BAC-Cre mice). **i** Spearman correlation analyses of VTA→BLA dopamine neuron firing activity with the social interaction behavior and with the time in EPM open arms after CSDS (p=0.66, p=0.16, n=16, 3-7 neurons per mouse, combined C57BL6/J and TH-BAC-Cre mice). In all panels, data are represented as mean ± s.e.m.; two-sided statistical analyses and post hoc tests were performed, *p<0.05.

Supplementary Fig. 4. VTA→BLA neuronal dynamic does not correlate with social interaction behaviors. a Schematic of the fiber photometry system used, two light-emitting diodes at 490 nm and 405 nm, reflected off dichroic mirrors, coupled into the optical fiber. Two output signals were projected onto a photodetector, after which they were separated for analysis and time locked with the video tracking software system. **b** Social interaction ratio showing no differences in basal social interaction behaviors between stress naïve mice not implanted and stress naïve mice implanted for fiber photometry (Mann Whitney test, U=138 p=0.73, n=15,20) and 3D representation of the z-scored GCamp6 signal, 405nm signal and mouse velocity upon the mouse position during the social interaction test with social target present. **c** Time (%) in EPM open arms showing no differences in basal anxiety-like behaviors between stress naïve mice not implanted and stress naïve mice implanted for fiber photometry (Mann Whitney test, U=111 p=0.17, n=15,20). **d** Without social target present. (Left) Sample trace (bars represent when the mouse is in the SI zone) and 3D representation of the GCamp6 ΔF/F upon the mouse position during the SI test without social target present. (Right) Pearson correlation analyses of mean SI zone VTA→BLA activity as mean ΔF/F z-score AUC (r^2=0.016 p=0.56, n=23) and number of events per minute (r^2=0.004 p=0.77, n=23) with the time in SI zone. **e** Same as **d** with social target present (AUC: r^2=0.02 p=0.5; events per minute: r^2=0.08 p=0.18, n=23 stress-naïve C57BL6/J mice). **f** Dynamics of averaged GCaMP6s and GCaMP6f signals and (Bottom) related 405nm signals (z-scores mean ± s.e.m.) 5 s before and 5 s after the mice enter in SI zone without social target present (blue) and with social target present (red) showing that VTA→BLA neuronal activity does not increase when stress naïve mice transit to SI zone, (onset) averaged GCaMP6 and

405 nm signal z-scores within a 1 s bin (-0.5 to +0.5 s) when stress naïve mice transit to SI zone (GCaMP6: Student *t*-test, t=0.20 p=0.84; 405 nm: Mann Whitney test, U=5018, p=0.19, Bars represent mean ± s.e.m. for n=101,111 epochs). **g** (Top) Dynamics of GCaMP6s signals and (Bottom) related 405nm signals (z-scores mean ± s.e.m.) 5 s before and 5 s after the mice enter in SI zone without social target present (blue) and with social target present (red) showing that VTA→BLA neuronal activity does not increase when stress naïve mice transit to SI zone, (onset) averaged signal z-scores within a 1 s bin (-0.5 to +0.5 s) when stress naïve mice transit to SI zone (t.test, GCaMP6s: t=0.41 p=0.87, t=1.51 p=0.92, Bars represent mean ± s.e.m. for n=74,84 epochs). **h** (Top) Dynamics of GCaMP6f signals and (Bottom) related 405nm signals (z-scores mean ± s.e.m.) 5s before and 5s after the mice enter in SI zone without social target present (blue) and with social target present (red) showing that VTA→BLA neuronal activity does not increase when stress naïve mice transit to SI zone, (onset) averaged signal z-scores within a 1 s bin (-0.5 to +0.5 s) when stress naïve mice transit to SI zone (t.test, GCaMP6f: t=0.48 p=0.54, 405nm: t=1.74 p=0.087, Bars represent mean ± s.e.m. for n=24,27 epochs). In all panels, data are represented as mean ± s.e.m.; two-sided statistical analyses were performed.

Supplementary Fig. 5. VTA→BLA neuronal dynamic correlates with anxiety-like behaviors. a-e Before CSDS. **a** Pearson correlation analyses of mean VTA→BLA activity as mean ΔF/F z-score AUC in EPM center and closed arms with the time in EPM center and closed arms (r^2=0.01 p=0.03, r^2=0.19 p=0.14, n=23 stress naïve C57BL6/J mice). **b** Pearson correlation analyses of VTA→BLA activity expressed as events per minute with the time (%) in EPM center and closed arms (r^2=0.02, r^2=0.21, p=0.19, p=0.02, n=23 C57BL6/J mice). **c** Dynamics of 405 nm signals (z-scores mean ± s.e.m.) 5 s before and 5 s after the mice enter in EPM center, closed and open arms; (onset) averaged 405 nm signal z-scores within a 1 s bin (-0.5 to +0.5 s) while the mice enter in EPM closed arms, center or open arms (ANOVA, $F_{2/276}$=1.798, p=0.17, bars represent mean ± s.e.m. for n=80,98,100 epochs). **d** (Right) Dynamics of GCaMP6s and (Left) related 405 nm signals (z-scores mean ± s.e.m.) 5 s before and 5 s after the mice enter in EPM center, closed and open arms (ANOVA, GCaMP6s: $F_{2,213}$=6.91, p=0.006, post hoc t=2.92 p=0.01, t=2.82

p=0.02; 405 nm: $F_{2,213}$=2.49, p=0.09, n=62,72,80 epochs). **e** (Right) Dynamics of GCaMP6f and (Left) related 405 nm signals (z-scores mean ± s.e.m.) 5 s before and 5 s after the mice enter in EPM center, closed and open arms (ANOVA, GCaMP6f: $F_{2,60}$=5.406, p=0.007, post hoc t=2.33 p=0.049, t=3.28 p=0.005; 405nm: $F_{2,60}$=0.014, p=0.68, n=20-25). **f-i** After CSDS. **f** Spearman correlation analyses of mean VTA→BLA activity as mean ΔF/F z-score AUC in EPM center and closed arms with the time in EPM open arms, center and closed arms after CSDS (CTL mice r=0.82 p=0.03, AD & A mice r=0.45, p=0.08, r=0.02, p=0.92, r=-0.3 p=0.16, n=23 mice). **g** Spearman correlation analyses of VTA→BLA activity with the time (%) in EPM open arms, center and closed arms (CTL mice r=0.14, p=0.78, AD & A mice r=0.51 p=0.04, r=0.34 p=0.10, r=-0.52 p=0.01, n=23 mice). **h** Dynamics of 405 nm signals (z-scores mean ± s.e.m.) 5 s before and 5 s after the socially stressed mice enter in EPM center, closed and open arms; (onset) averaged 405 nm signal z-scores within a 1 s bin (-0.5 to +0.5 s) while the mice enter in EPM closed arms, center or open arms (ANOVA, $F_{2,188}$=2.538, p=0.082, bars represent mean ± s.e.m. for n=46,65,75 epochs). **i** Dynamics of 405nm signals (z-scores mean ± s.e.m.) 5 s before and 5 s after the control mice enter in EPM center, closed and open arms (ANOVA, $F_{2,92}$=1.203, p=0.30, Bars represent mean ± s.e.m. for n=30,30,35 number of trials)**.** In all panels, data are represented as mean ± s.e.m.; two-sided statistical analyses and post hoc corrected tests were performed, *p<0.05, **p<0.01.

Supplementary Fig. 6. NpHR optogenetic modulation of VTA→BLA neurons induces anxiety-like behaviors. a Schematic of the VTA→BLA brain surgery in C57BL6/J mice, immunohistochemistry validation (scale bar, 100 μm) and quantification of 83% colocalization (3-4 sections per mouse from 3 mice). **b-c** Supplementary results of mouse behaviors depicted in Fig. 4. **b** NpHR optogenetic modulation ON of Sub.D-NpHR and Sub.D-eYFP mice (respective Mann-Whitney or t-test analysis analysis, Number of EPM open arms entries: U=20 p=0.33; Time (s) in EPM open arms: t=2.333 p=0.032, closed arms: t=3.65 p=0.002, center: t=0.927 p=0.37, velocity**:** t=0.916 p=0.37, and locomotion: t=1.028 p=0.32). **c** Same as **b** while NpHR optogenetic modulation OFF (respective Mann-Whitney or t-test analysis, Number of EPM open arms entries: t=1.124 p=0.27, Time (s) in EPM open arms: t=0.684 p=0.50, closed arms: t=0.863 p=0.40,

center: t=0.040 p=0.93, velocity: U=33 p=0.35, locomotion: U=31 p=0.28). In all panels, data are represented as mean ± s.e.m., n=9, 10 mice; two-sided statistical analyses were performed, *p<0.05, **p<0.01.

Supplementary Fig. 7. NpHR optogenetic modulation of VTA→BLA neurons induces anxiety-like behaviors in stress-naïve control mice.
a Timeline and schematic of the behavioral experiment in stress-naïve CTL C57BL6/J mice. **b** NpHR optogenetic modulation ON: 0.1Hz 5s pulse width, in CTL-NpHR and CTL-eYFP mice (respective Mann-Whitney or t-test analyses, Time (%) in EPM open arms: t=0.97 p=0.38, Number of EPM open arms entries: t=1.39 p=0.19, Time (s) in EPM open arms: t=0.29 p=0.79, closed arms: U=26 p=0.69, center: t=0.58 p=0.57, velocity**:** t=1.68 p=0.07, locomotion: t=1.593 p=0.13). **c** Same as **b** while NpHR optogenetic modulation OFF (respective Mann-Whitney or t-test analysis, Time (%) in EPM open arms: U=16 p=0.33, Number of EPM open arms entries: U=20 p=0.67, Time (s) in EPM open arms: U=17 p=0.33, closed arms: U=14 p=0.23, center: t=0.38 p=0.79, velocity: t=1.4 p=0.15, locomotion: t=0.25 p=0.61). Bars represent mean ± s.e.m. for n=8,7 number of mice**.**
d Separate cohort of stress-naïve CTL C57BL6/J mice and NpHR mice exposed to optogenetic modulation ON during the 300 s EPM trial of CTL-NpHR and CTL-eYFP mice (respective Mann-Whitney or t-test analyses, Time (%) in EPM open arms: t=3.029, p=0.007, Number of EPM open arms entries: t=1.19 p=0.25, Time (s) in EPM open arms: t=3.029 p=0.007, closed arms: t=1.993 p=0.06, center: t=1.509 p=0.14, velocity**:** U=36 p=0.31, locomotion: U=24 p=0.052, n=10,10 mice). **e** Same as **d** while NpHR optogenetic modulation OFF (respective Mann-Whitney or t-test analysis, Time (%) in EPM open arms: t=0.17 p=0.86, Number of EPM open arms entries: t=0.95 p=0.35, Time (s) in EPM open arms: U=42 p=0.57, closed arms: t=0.95 p=0.35, center: t=1.37 p=0.18, velocity: U=45 p=0.73, locomotion: U=39 p=0.43, n=10,10 number of mice**.** In all panels, data are represented as mean ± s.e.m.; two-sided statistical analyses were performed, *p<0.05, **p<0.01.

Supplementary Fig. 8. NpHR optogenetic modulation of VTA→BLA neurons induces transient anxiety-like behaviors but not depressive-like behaviors. a

Experimental timeline in a separate cohort of C57BL6/J mice. **b** NpHR optogenetic modulation ON -0.1Hz 5 s pulse width, in Sub.D-NpHR and Sub.D-eYFP mice, n=11,12 number of mice, performing a social interaction, female urine sniffing, EPM and OFT tests (t=0.55 p=0.52, t=0.57 p=0.56, t=0.79 p=0.44, t=0.20 p=0.84, t=2.619 p=0.042, t=1.826 p=0.06, t=1.97 p=0.53, t=2.087 p=0.049, t=0.69 p=0.49, t=0.68 p=0.50). **c** Same as **b** while NpHR optogenetic modulation OFF (t=0.88 p=0.44, t=0.067 p=0.94, t=0.78 p=0.44, t=0.27 p=0.77, t=0.19 p=0.87, t=0.27 p=0.78, t=0.14 p=0.82, U=36 p=0.069, U=63 p=0.88, t=0.77 p=0.44). **d** Sucrose preference and Time (%) in EPM open arms 7 days after the last NpHR optogenetic modulation (respective analysis, t=0.1228 p=0.90, t=1.313 p=0.72, p>0.05). In all panels, data are represented as mean ± s.e.m.; two-sided Mann-Whitney or t-test statistical analyses were performed, *p<0.05.

Supplementary Fig. 9. ChR2 optogenetic stimulation of VTA→BLA neurons reduces anxiety-like behaviors. a Schematic of the VTA→BLA brain surgery in C57BL6/J mice, immunohistochemistry validation (scale bar, 100 µm) and quantification of 82% colocalization (3-4 sections per mouse from 3 mice). **b-g** Supplementary analysis of mouse behaviors depicted in Fig. 4. **b** Social interaction behaviors following CSDS (ANOVA, $F_{2/60}$=19.37 p=6.05e-07; post hoc test corrected p-values, t=5.53 p=0.001; t=4.94 p=0.001, t=0.089 p=0.93, CTL n=25, AD n=18 and A n=17 mice). **c** Time (%) spent in open arms in AD mice: (Left) EPM test while ChR2 optogenetic stimulation is OFF, (Middle) all trial ChR2 optogenetic stimulation; (Right) ChR2 optogenetic stimulation selective to the EPM center (t-tests: t=0.21 p=0.84, t=2.13 p=0.49, t=2.81 p=0.02, n=9, 9). **d** Same, as **c** in A mice (t-test and Mann-Whitney test: t=0.067 p=0.94, U=47 p=0.018, t=2.81 p=0.017, n=8, 9). **e** EPM test while ChR2 optogenetic stimulation is OFF in CSDS-eYFP mice and CSDS-ChR2 mice (respective analysis, Number of EPM open arms entries: t=0.71 p=0.48, Time (s) in EPM open arms: t-tests, t=0.09 p=0.96, closed arms: t=0.65 p=0.5, center: t=0.513 p=0.67, velocity: t=0.20 p=0.99, locomotion: t=0.30 p=0.98, p>0.05, n=17, 19). **f** Same as **e** while during all trial ChR2 optogenetic stimulation (respective t-tests analysis, t=2.94 p=0.006, t=3.38 p=0.002, t=2.30 p=0.03, t=3.6 p=0.001, t=1.69 p=0.28; t=1.99 p=0.16, n=17, 19 number of mice). **g** Same as **e** while during ChR2 optogenetic stimulation selective to the EPM center (respective t-tests

analysis, t=3.94 p=0.006, t=3.66 p=0.0008, t=2.75 p=0.009; t=3.77 p=0.007; t=1.10 p=0.62; t=0.86 p=0.77, n=17, 19). In all panels, data are represented as mean ± s.e.m. for n number of mice; two-sided statistical analyses and post hoc corrected tests were performed, *p<0.05, **p<0.01, ***p<0.001.

Supplementary Fig. 10. ChR2 optogenetic stimulation of VTA→BLA neurons rescues anxiety-like behaviors but not depressive-like behaviors. a Experimental timeline in a separate cohort of C57BL6/J mice. **b** Social interaction behaviors, preference for female urine, time (%) in EPM open arms, time (%) in OFT center, velocity and locomotion in AD-ChR2 and AD-eYFP mice before optogenetic stimulation (t=1.06 p=0.30, t=0.577 p=0.57, t=0.20 p=0.83, t=0.348 p=0.73, t=1.67 p=0.11, t=1.178 p=0.26, t=0.978 p=0.34). **b'** Social interaction behaviors, preference for female urine, time (%) in EPM open arms, time (%) in OFT center, velocity and locomotion in AD-ChR2 and AD-eYFP mice during ChR2 optogenetic stimulation (t=0.78 p=0.44, t=0.07 p=0.93, t=2.430 p=0.031, t=3.81 p=0.001, t=2.152 p=0.47 , t=1.158 p=0.26, t=1.135 p=0.27). **c** Social interaction behaviors, preference for female urine, time (%) in EPM open arms, time (%) in OFT center, velocity and locomotion in A-ChR2 and A-eYFP mice before optogenetic stimulation (t=0.94 p=0.36, t=0.91 p=0.38, t=0.53 p=0.60, t=0.54 p=0.59, t=0.13 p=0.89, t=1.89 p=0.06, t=1.55 p=0.15). **c'** Social interaction behaviors, preference for female urine, time (%) in EPM open arms, time (%) in OFT center, velocity and locomotion in A-ChR2 and A-eYFP mice during ChR2 optogenetic stimulation (t=0.397 p=0.69, t=1.30 p=0.22, t=2.408 p=0.04, t=2.21 p=0.047, t=0.28 p=0.61, t=0.72 p=0.48, t=0.889 p=0.39). **d** Sucrose preference and Time (%) in EPM open arms 7 days after the last optogenetic modulation in AD-ChR2 and AD-eYFP mice (t=075 p=0.46, U=31, p=0.72) and **e** Sucrose preference and Time (%) in EPM open arms 7 days after the last optogenetic modulation in A-ChR2 and A-eYFP mice (U=15.5 p=0.5, t=0.83, p=0.42, n=6, 9 mice). In all panels, data are represented as mean ± s.e.m.; t-test or Mann-Whitney two-sided statistical analyses were performed, *p<0.05.

Supplementary Fig. 11. ChR2 optogenetic stimulation of VTA→BLA neurons in stress naïve mice. a Timeline of ChR2 optogenetic experiments performed in stress

naïve CTL C57BL6/J mice. **b** EPM test while ChR2 optogenetic stimulation is OFF in stress-naïve CTL-eYFP mice and CTL-ChR2 mice (respective analysis, Time (%) in EPM open arms in CTL-ChR2 and CTL-eYFP mice, t-test, t=0.28, p=0.77, Number of EPM open arms entries: t=0.54 p=0.93, Time (s) in EPM open arms: t=0.07 p=0.99, closed arms: t=0.76 p=0.84, center: t=0.99 p=0.69, velocity: t=0.11 p=0.99, locomotion: t=0.22 p=0.99, n=12, 13). **c** Same as **b** during all trial ChR2 optogenetic stimulation (respective analysis, Time (%) in EPM open arms in CTL-ChR2 and CTL-eYFP mice, t=0.66, p=0.51, Number of EPM open arms entries: t=1.63 p=0.33, Time (s) in EPM open arms: t=1.79 p=0.25, closed arms: t=0.26 p=0.99, center: t=0.05 p=0.99, velocity: t=0.18 p=0.99, locomotion: t=0.004, p=0.99, n=8, 6). **d** Same as **c** during ChR2 optogenetic stimulation selective to the EPM center (respective analysis, Time (%) in EPM open arms in CTL-ChR2 and CTL-eYFP mice, t-test: t=1.77 p=0.09, Number of EPM open arms entries t=2.19 p=0.13, Time (s) in EPM open arms: t=1.55 p=0.36, closed arms: t=0.93 p=0.74, center: t=1.07 p=0.65, velocity: t=0.11 p=0.99, locomotion: t=0.48, p=0.95, n=8, 11). **e** Experimental timeline in a separate cohort of stress-naïve CTL C57BL6/J mice. **f** Social interaction behaviors, preference for female urine, time (%) in EPM open arms, time (%) in OFT center, velocity and locomotion in CTL-ChR2 and CTL-eYFP mice before optogenetic stimulation (respective analyses for n=9, 8 mice, t=0.275 p=0.78, U=21.5 p=0.176, t=1.43 p=0.16, t=0.62 p=0.545, t=0.24 p=0.81, t=0.25 p=0.80, t=0.75 p=0.46). **g** Social interaction behaviors, preference for female urine, time (%) in EPM open arms, time (%) in OFT center, velocity and locomotion in CTL-ChR2 and CTL-eYFP mice during ChR2 optogenetic stimulation (respective analyses, t=0.55 p=0.59, U=34 p=0.88, t=0.38 p=0.71, t=0.88 p=0.38, t=0.89 p=0.39, t=0.62 p=0.51, U=35, p=0.67, n=9, 8 mice). **h** Sucrose preference and Time (%) in EPM open arms 7 days after the last optogenetic modulation (respective analyses, t=0.81 p=0.43, t=1.251, p=0.22 for n=9, 8 mice). In all panels, data are represented as mean ± s.e.m.; two-sided statistical analyses were performed.

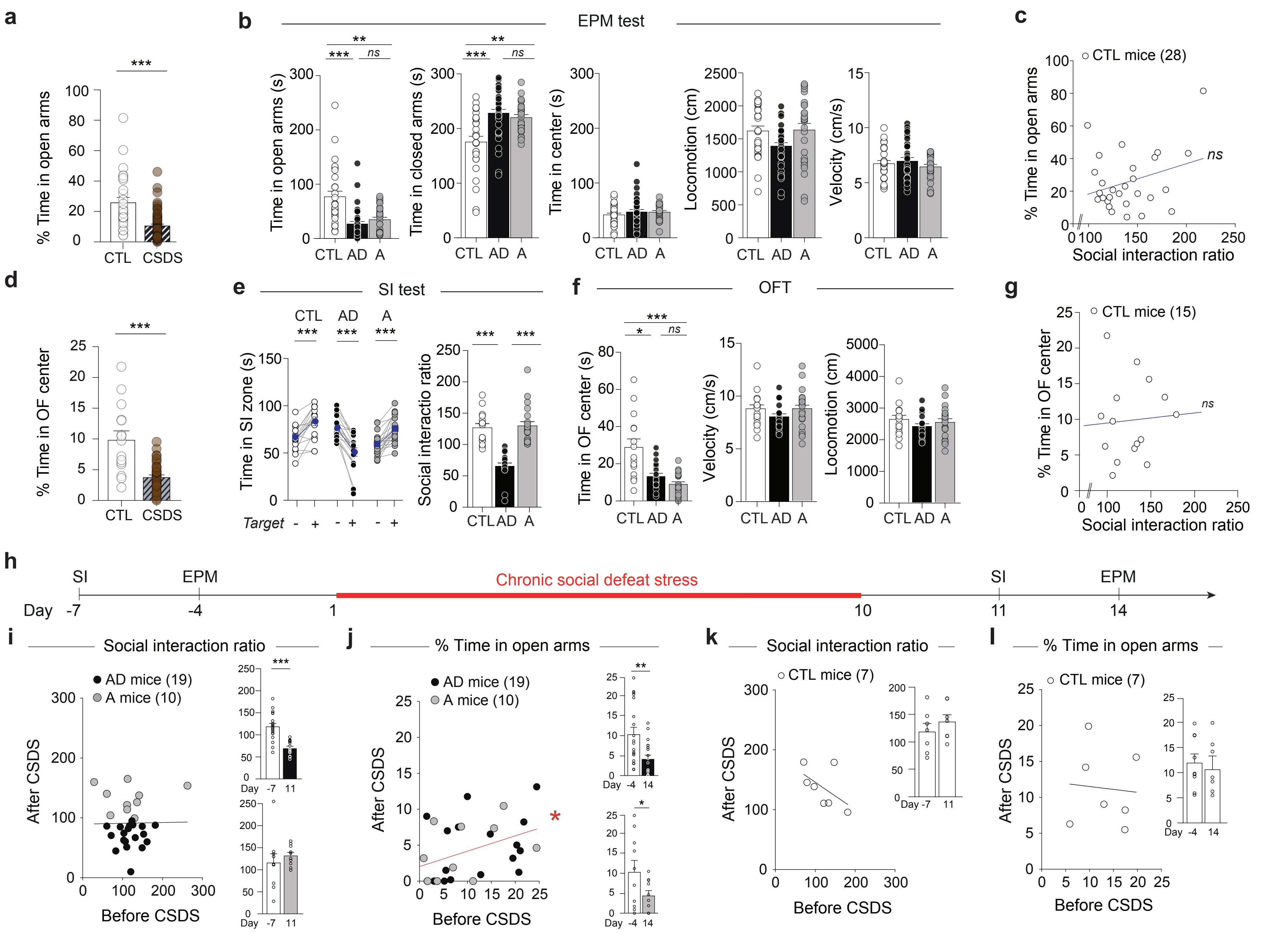
a
b
EPM test
c
% Time in open arms
CTL CSDS
Time in open arms (s)
Time in closed arms (s)
Time in center (s)
Locomotion (cm)
Velocity (cm/s)
CTL AD A
ns
CTL mice (28)
% Time in open arms
Social interaction ratio
d
% Time in OF center
e
SI test
CTL AD A
Time in SI zone (s)
Target - + - + - +
Social interactio ratio
f
OFT
Time in OF center (s)
Velocity (cm/s)
Locomotion (cm)
g
CTL mice (15)
% Time in OF center
Social interaction ratio
h
SI
EPM
Chronic social defeat stress
SI
EPM
Day -7 -4 1 10 11 14
i
Social interaction ratio
AD mice (19)
A mice (10)
After CSDS
Before CSDS
j
% Time in open arms
AD mice (19)
A mice (10)
After CSDS
Before CSDS
k
Social interaction ratio
CTL mice (7)
After CSDS
Before CSDS
l
% Time in open arms
CTL mice (7)
After CSDS
Before CSDS

Sup. Figure 1

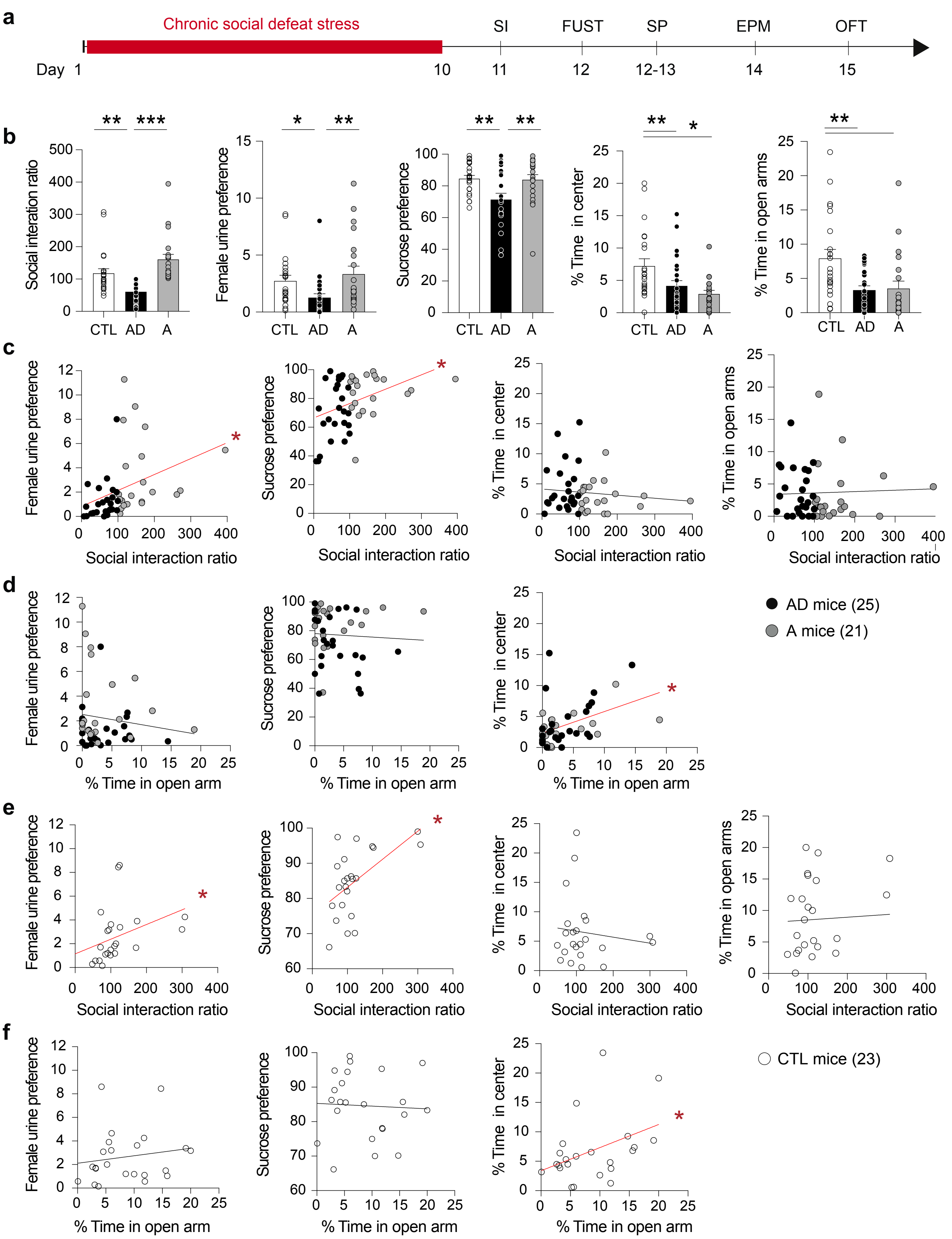
a
Chronic social defeat stress
SI
FUST
SP
EPM
OFT
Day 1
10
11
12
12-13
14
15
b
Social interaction ratio
Female urine preference
Sucrose preference
% Time in center
% Time in open arms
CTL
AD
A
c
Social interaction ratio
d
% Time in open arm
AD mice (25)
A mice (21)
e
f
CTL mice (23)

Sup. Figure 2

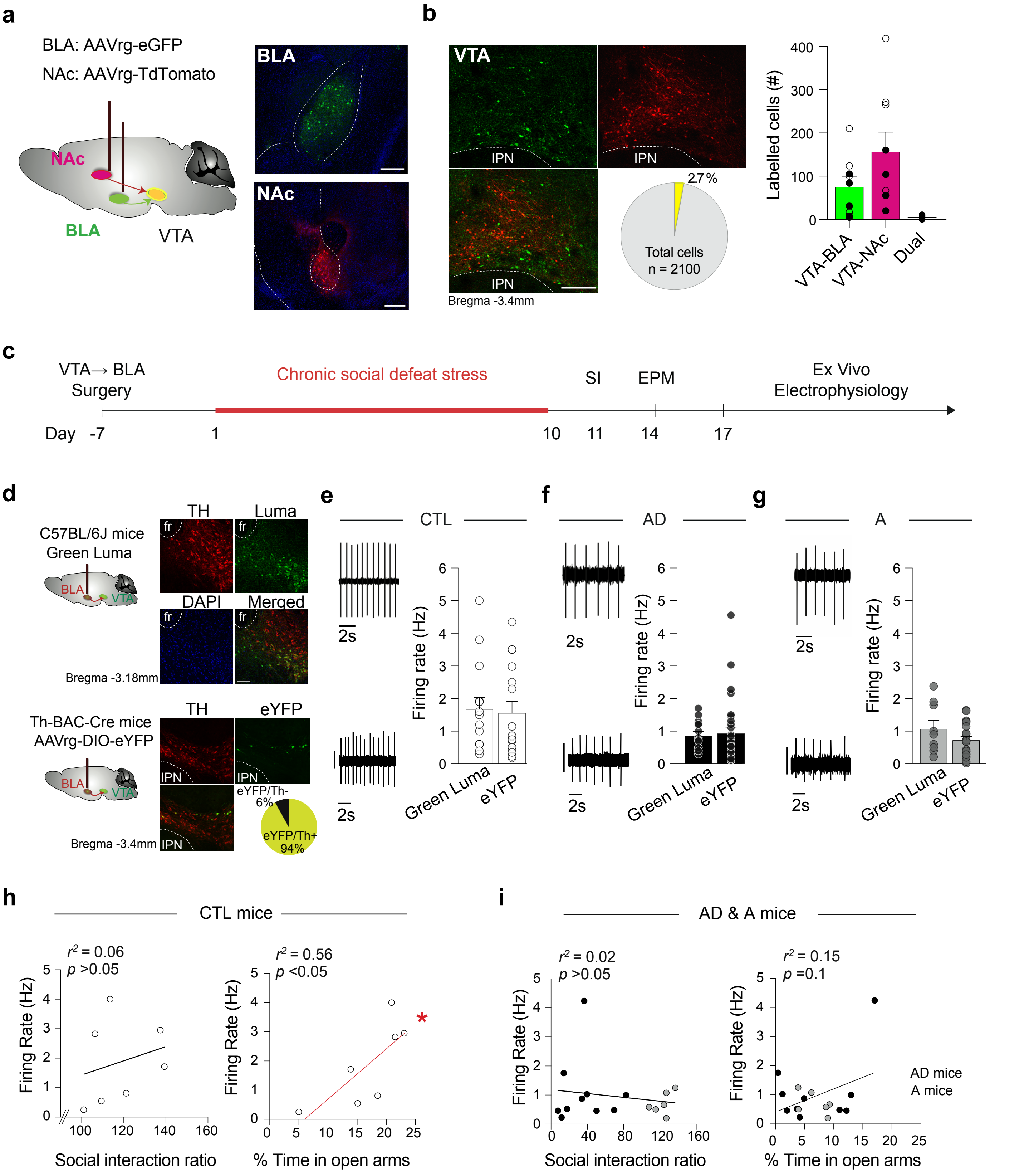
a
BLA: AAVrg-eGFP
NAc: AAVrg-TdTomato
NAc
BLA
VTA
b
VTA
IPN
Bregma -3.4mm
2.7 %
Total cells
n = 2100
Labelled cells (#)
VTA-BLA
VTA-NAc
Dual
c
VTA→ BLA
Surgery
Chronic social defeat stress
SI
EPM
Ex Vivo
Electrophysiology
Day -7
1
10
11
14
17
d
C57BL/6J mice
Green Luma
TH
Luma
DAPI
Merged
fr
Bregma -3.18mm
Th-BAC-Cre mice
AAVrg-DIO-eYFP
eYFP
eYFP/Th-
6%
eYFP/Th+
94%
Bregma -3.4mm
e
CTL
f
AD
g
A
2s
Firing rate (Hz)
Green Luma
eYFP
h
CTL mice
r^2 = 0.06
p >0.05
r^2 = 0.56
p <0.05
Firing Rate (Hz)
Social interaction ratio
% Time in open arms
i
AD & A mice
r^2 = 0.02
p >0.05
r^2 = 0.15
p =0.1
AD mice
A mice

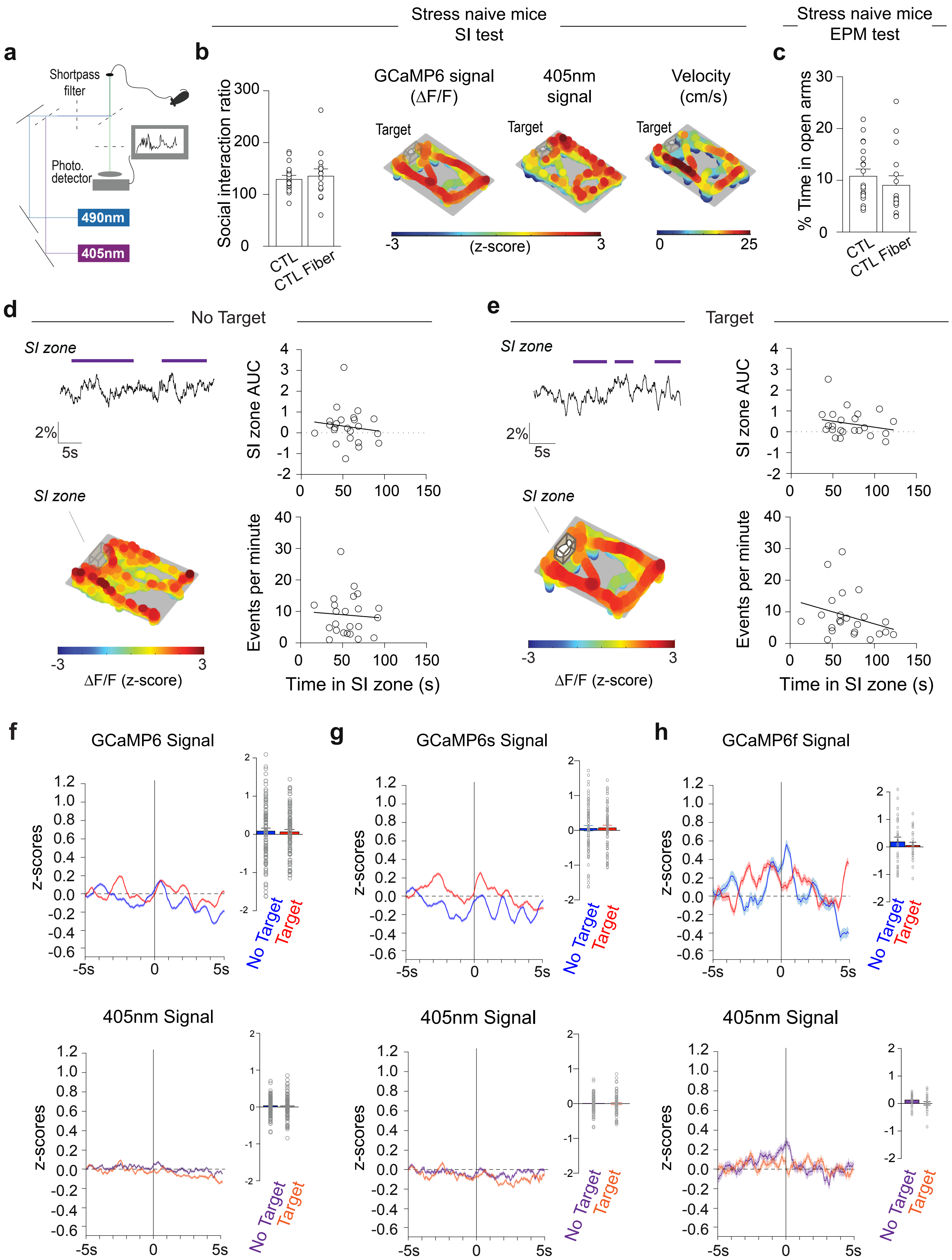
Stress naive mice
SI test
Stress naive mice
EPM test
a
Shortpass filter
Photo. detector
490nm
405nm
b
Social interaction ratio
CTL
CTL Fiber
GCaMP6 signal (ΔF/F)
405nm signal
Velocity (cm/s)
Target
(z-score)
c
% Time in open arms
CTL
CTL Fiber
d
No Target
SI zone
2%
5s
SI zone AUC
Events per minute
Time in SI zone (s)
ΔF/F (z-score)
e
Target
SI zone
2%
5s
SI zone AUC
Events per minute
Time in SI zone (s)
ΔF/F (z-score)
f
GCaMP6 Signal
z-scores
No Target
Target
405nm Signal
g
GCaMP6s Signal
z-scores
No Target
Target
405nm Signal
h
GCaMP6f Signal
z-scores
No Target
Target
405nm Signal

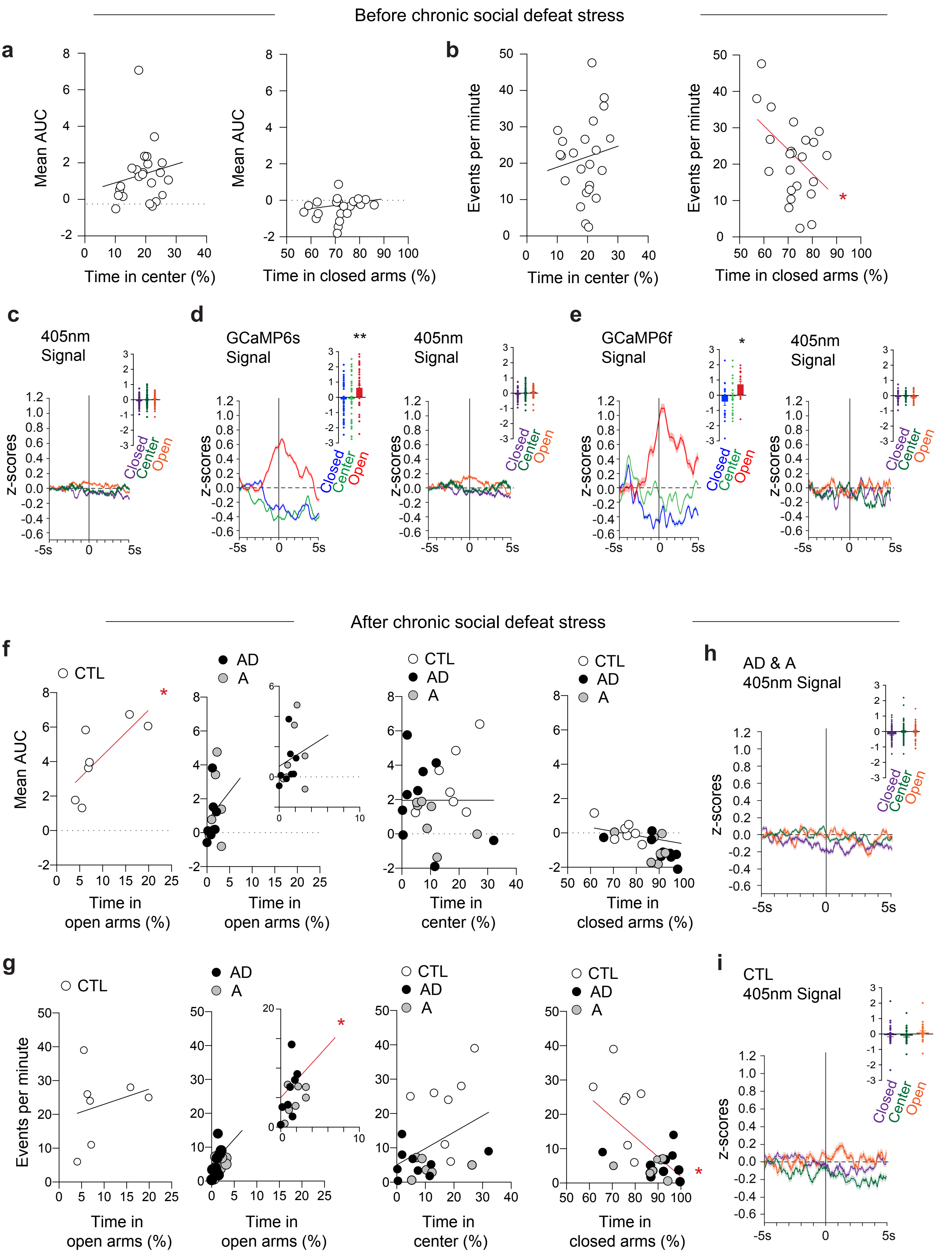
Before chronic social defeat stress
a
Mean AUC
Time in center (%)
Time in closed arms (%)
b
Events per minute
Time in center (%)
Time in closed arms (%)
*
c
405nm Signal
d
GCaMP6s Signal
**
405nm Signal
e
GCaMP6f Signal
*
405nm Signal
z-scores
Closed
Center
Open
-5s
0
5s
After chronic social defeat stress
f
CTL
AD
A
Mean AUC
Time in open arms (%)
Time in center (%)
Time in closed arms (%)
*
h
AD & A 405nm Signal
g
Events per minute
*
i
CTL 405nm Signal

Sup. Figure 5

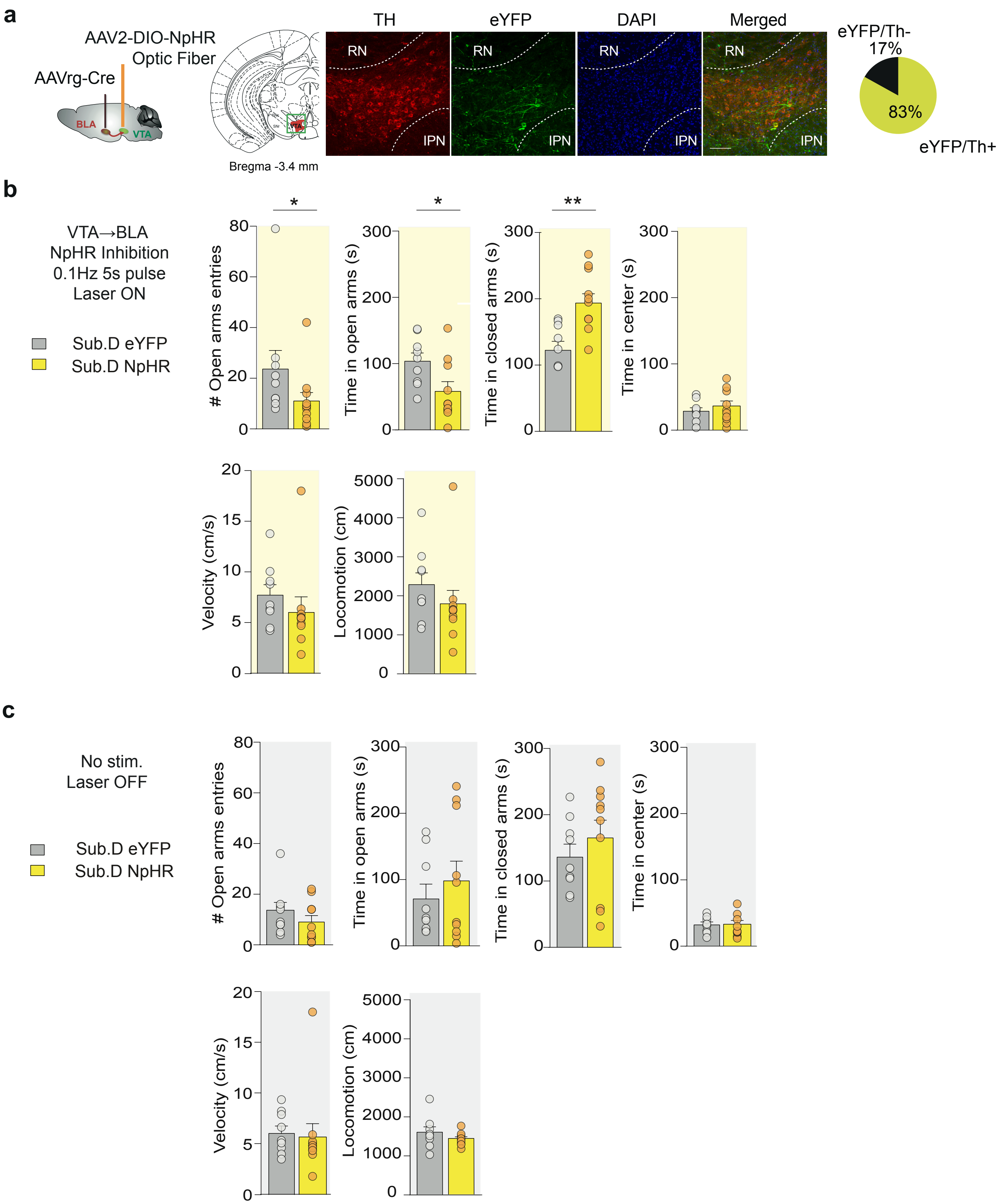
a
AAV2-DIO-NpHR
Optic Fiber
AAVrg-Cre
BLA
VTA
Bregma -3.4 mm
TH
eYFP
DAPI
Merged
RN
IPN
eYFP/Th-
17%
83%
eYFP/Th+
b
VTA→BLA
NpHR Inhibition
0.1Hz 5s pulse
Laser ON
Sub.D eYFP
Sub.D NpHR
Open arms entries
Time in open arms (s)
Time in closed arms (s)
Time in center (s)
Velocity (cm/s)
Locomotion (cm)
c
No stim.
Laser OFF
Sub.D eYFP
Sub.D NpHR
Open arms entries
Time in open arms (s)
Time in closed arms (s)
Time in center (s)
Velocity (cm/s)
Locomotion (cm)

Sup. Figure 6

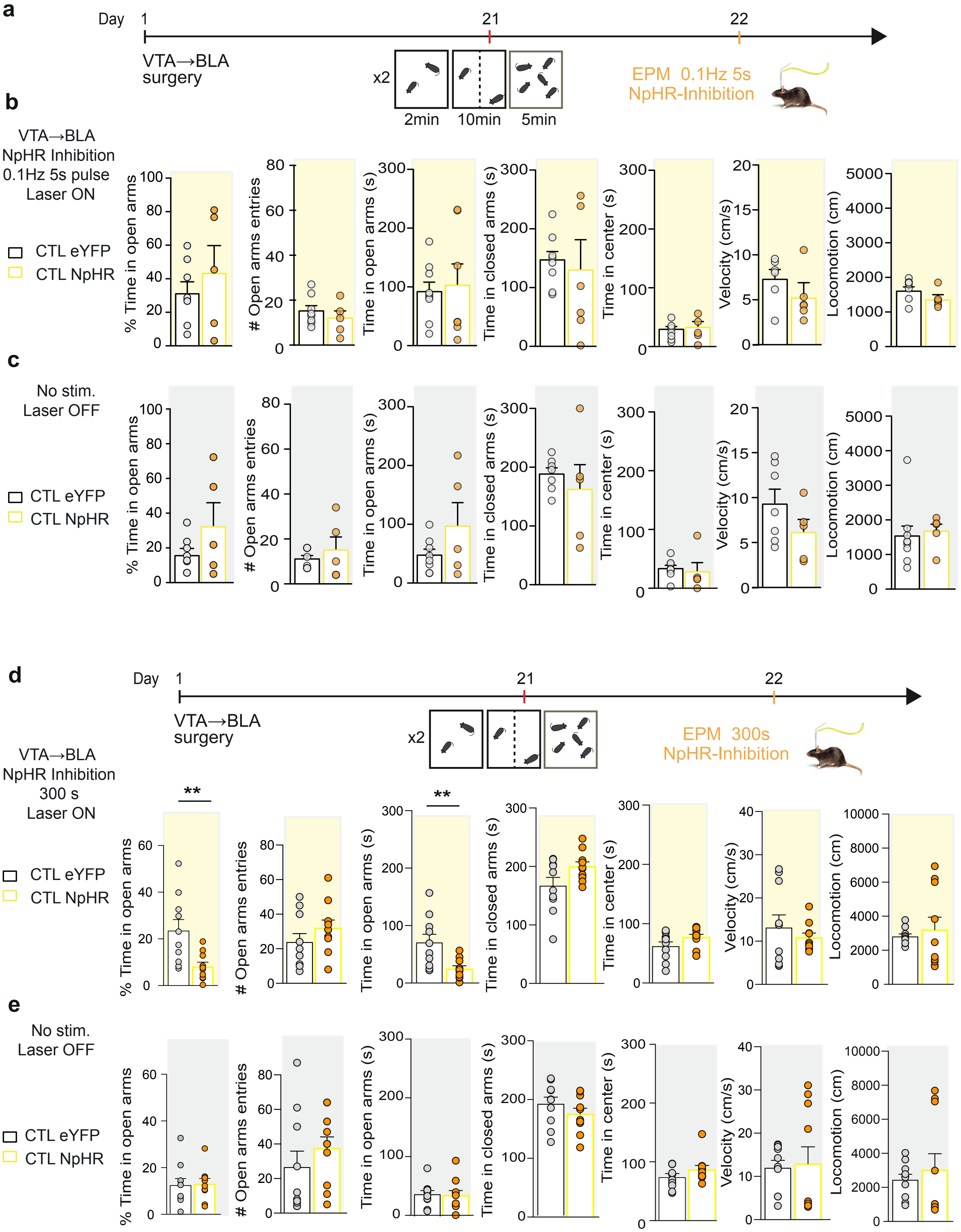
a
Day
1
21
22
VTA→BLA surgery
x2
2min
10min
5min
EPM 0.1Hz 5s NpHR-Inhibition
b
VTA→BLA NpHR Inhibition 0.1Hz 5s pulse Laser ON
CTL eYFP
CTL NpHR
% Time in open arms
# Open arms entries
Time in open arms (s)
Time in closed arms (s)
Time in center (s)
Velocity (cm/s)
Locomotion (cm)
c
No stim. Laser OFF
CTL eYFP
CTL NpHR
d
Day
1
21
22
VTA→BLA surgery
x2
EPM 300s NpHR-Inhibition
VTA→BLA NpHR Inhibition 300 s Laser ON
CTL eYFP
CTL NpHR
**
**
e
No stim. Laser OFF
CTL eYFP
CTL NpHR

Sup. Figure 7

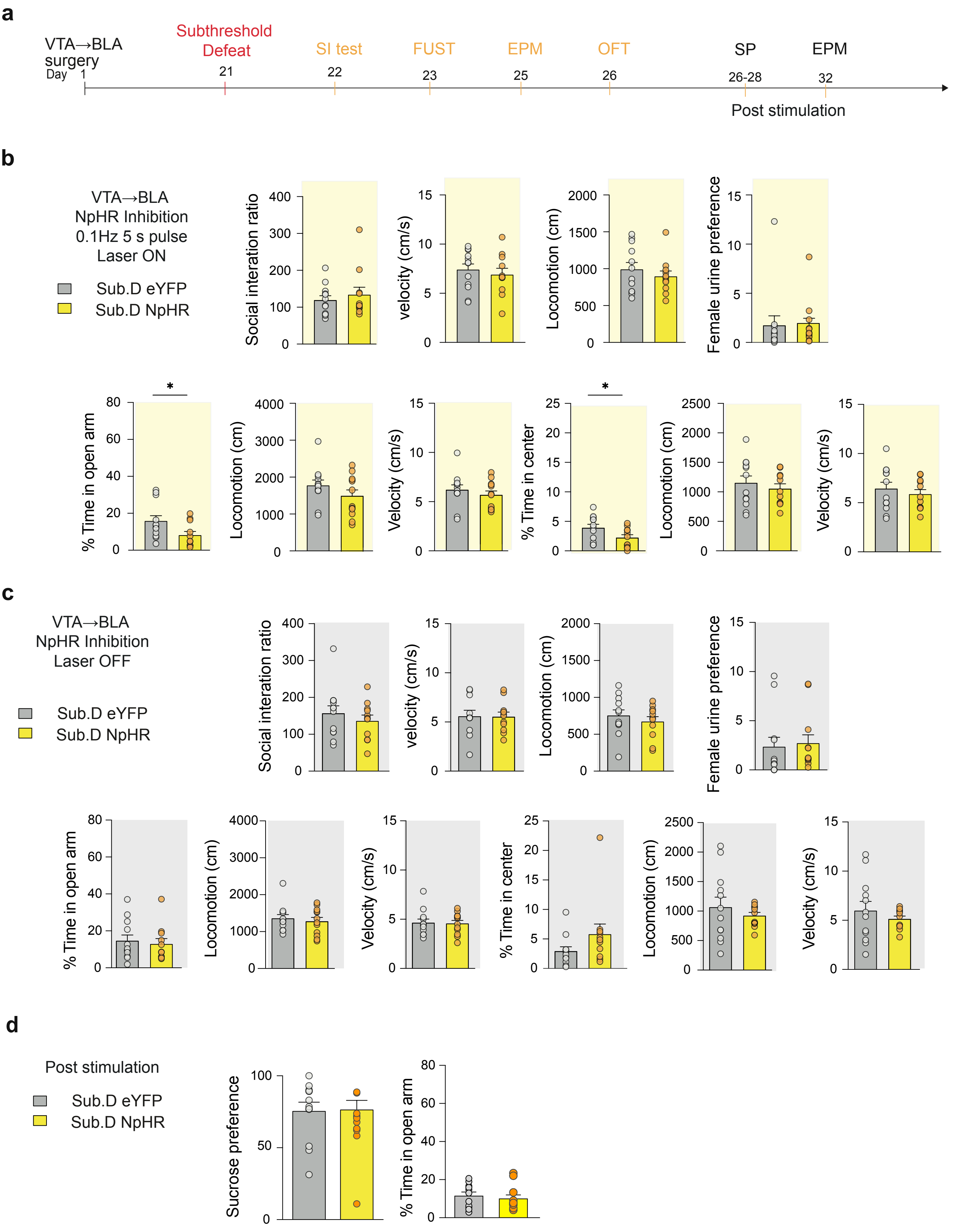

a
VTA→BLA surgery
Day 1
Subthreshold Defeat
21
SI test
22
FUST
23
EPM
25
OFT
26
SP
26-28
EPM
32
Post stimulation
b
VTA→BLA NpHR Inhibition 0.1Hz 5 s pulse Laser ON
Sub.D eYFP
Sub.D NpHR
Social interation ratio
velocity (cm/s)
Locomotion (cm)
Female urine preference
% Time in open arm
Velocity (cm/s)
% Time in center
c
VTA→BLA NpHR Inhibition Laser OFF
Sub.D eYFP
Sub.D NpHR
d
Post stimulation
Sub.D eYFP
Sub.D NpHR
Sucrose preference
% Time in open arm

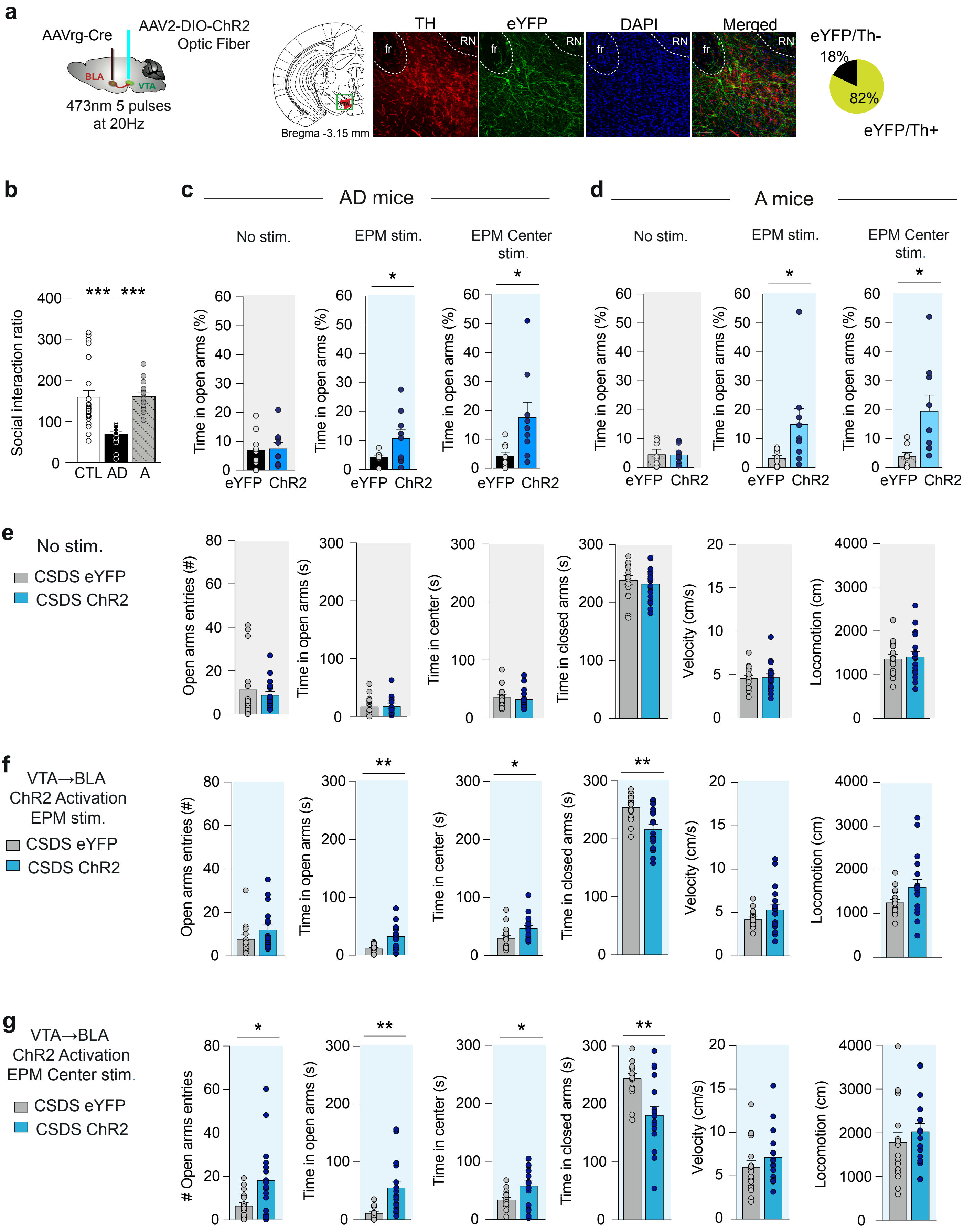
a
AAVrg-Cre
AAV2-DIO-ChR2
Optic Fiber
BLA
VTA
473nm 5 pulses
at 20Hz
Bregma -3.15 mm
TH
eYFP
DAPI
Merged
fr
RN
eYFP/Th-
18%
82%
eYFP/Th+
b
Social interaction ratio
CTL AD A
c
AD mice
No stim.
EPM stim.
EPM Center stim.
Time in open arms (%)
eYFP ChR2
d
A mice
e
No stim.
CSDS eYFP
CSDS ChR2
Open arms entries (#)
Time in open arms (s)
Time in center (s)
Time in closed arms (s)
Velocity (cm/s)
Locomotion (cm)
f
VTA→BLA
ChR2 Activation
EPM stim.
g
VTA→BLA
ChR2 Activation
EPM Center stim.
Open arms entries

Sup. Figure 9

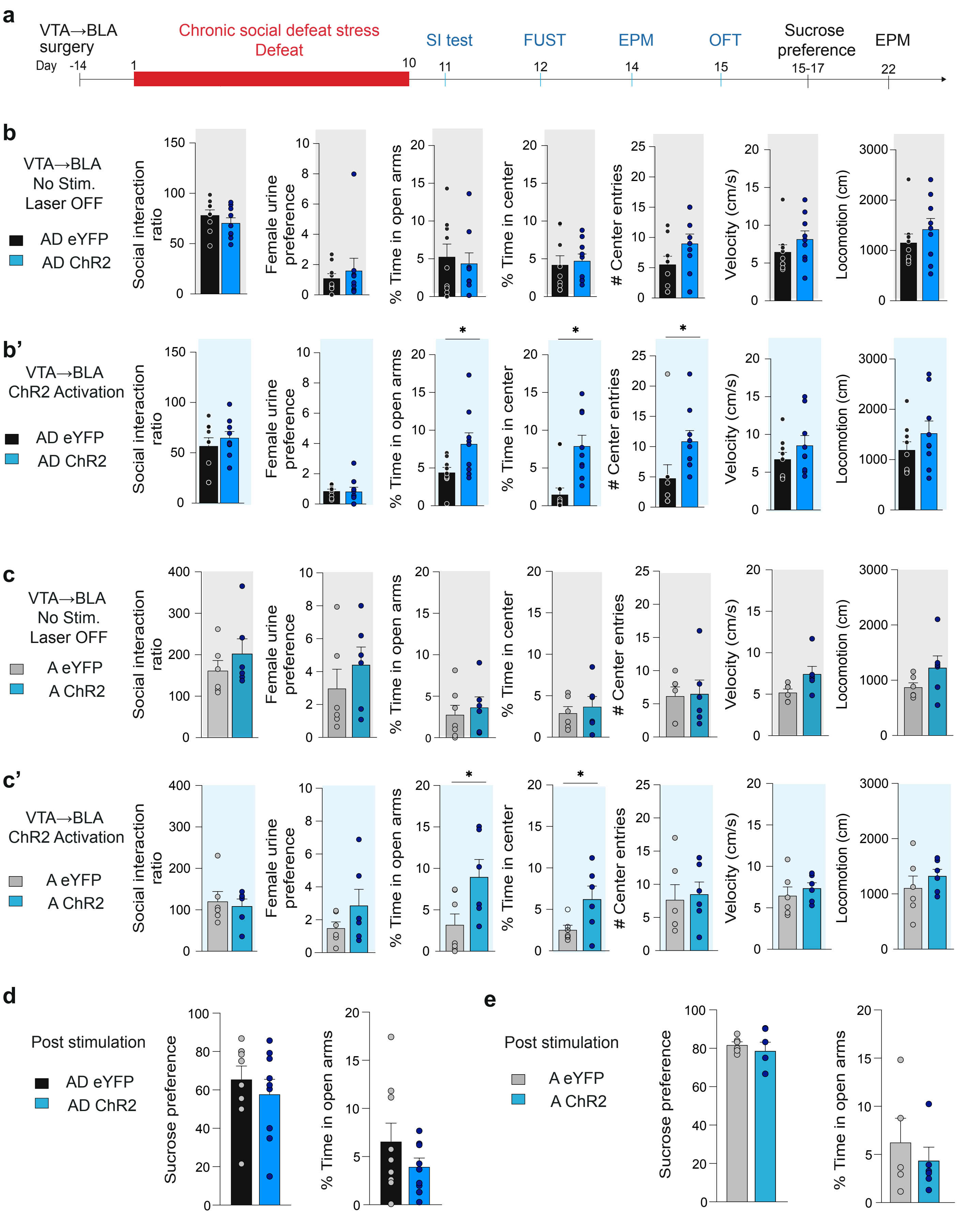

Sup. Figure 10

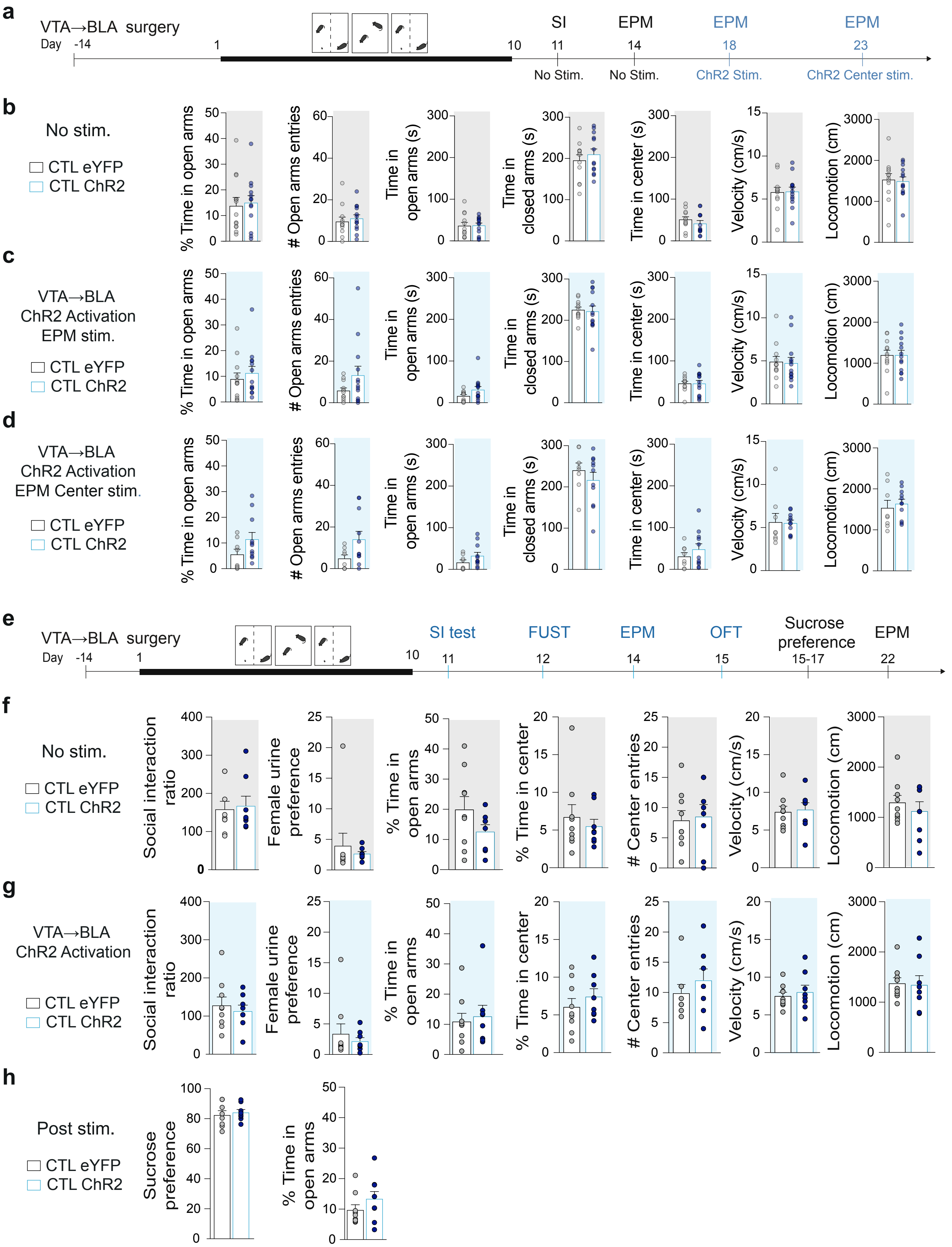

Sup. Figure 11
