# Supplementary material for: Midbrain projection to the basolateral amygdala encodes anxiety-like but not depression-like behaviors
Source: Nat Commun. 2022 Mar 22;13:1532. doi: 10.1038/s41467-022-29155-1 (PMC8940900; doi:10.1038/s41467-022-29155-1)
Supplement: Supplementary file 3 — Description of Additional Supplementary Files [file 41467_2022_29155_MOESM3_ESM.pdf]

Midbrain projection to the basolateral amygdala encodes

anxiety-like but not depression-like behaviors

Inventory of Supporting Information: NCOMMS-20-44686B

1 **Main**

Title
Author list
Affiliations
Abstract
Introduction
Results
Discussion
Methods
References
Acknowledgments
Author Contributions Statement
Competing Interests Statement
Figure Legends

4 **Figures**

15 **Supplementary Information**

1 *Supplementary Figure Legends*
11 *Supplementary Figures*
2 *Data Source Files*
1 *Reporting Summary*

1 **Response to Reviewer's comments**

1 **Editorial Policy Checklist**

1 **Author Checklist**
