# Supplementary material for: Midbrain projection to the basolateral amygdala encodes anxiety-like but not depression-like behaviors
Source: Nat Commun. 2022 Mar 22;13:1532. doi: 10.1038/s41467-022-29155-1 (PMC8940900; doi:10.1038/s41467-022-29155-1)
Supplement: Supplementary file 4 — Reporting Summary [file 41467_2022_29155_MOESM4_ESM.pdf]

nature portfolio

Corresponding author(s): Ming-Hu Han
Carole Morel

Last updated by author(s): Feb 7, 2022

Reporting Summary

Nature Portfolio wishes to improve the reproducibility of the work that we publish. This form provides structure for consistency and transparency in reporting. For further information on Nature Portfolio policies, see our Editorial Policies and the Editorial Policy Checklist.

Statistics

For all statistical analyses, confirm that the following items are present in the figure legend, table legend, main text, or Methods section.

n/a	Confirmed	
☐	☒	The exact sample size (*n*) for each experimental group/condition, given as a discrete number and unit of measurement
☐	☒	A statement on whether measurements were taken from distinct samples or whether the same sample was measured repeatedly
☐	☒	The statistical test(s) used AND whether they are one- or two-sided<br>*Only common tests should be described solely by name; describe more complex techniques in the Methods section.*
☐	☒	A description of all covariates tested
☐	☒	A description of any assumptions or corrections, such as tests of normality and adjustment for multiple comparisons
☐	☒	A full description of the statistical parameters including central tendency (e.g. means) or other basic estimates (e.g. regression coefficient) AND variation (e.g. standard deviation) or associated estimates of uncertainty (e.g. confidence intervals)
☐	☒	For null hypothesis testing, the test statistic (e.g. *F*, *t*, *r*) with confidence intervals, effect sizes, degrees of freedom and *P* value noted<br>*Give P values as exact values whenever suitable.*
☒	☐	For Bayesian analysis, information on the choice of priors and Markov chain Monte Carlo settings
☒	☐	For hierarchical and complex designs, identification of the appropriate level for tests and full reporting of outcomes
☐	☒	Estimates of effect sizes (e.g. Cohen's *d*, Pearson's *r*), indicating how they were calculated

Our web collection on statistics for biologists contains articles on many of the points above.

Software and code

Policy information about availability of computer code

Data collection	Behavioral measurements were collected via Ethovision XT11 (Noldus). Electrophysiological sample values were collected using a Digidata 1440A digitizer and pClamp 10.2 (Axon Instruments). A real-time fiber photometry signals were collected using signal processer (Tucker-Davis Technologies) and acquired with open source OpenEx software 2.20 controlling an RX8 lock-in amplifier (Tucker-Davis Technologies). Open-Ex (https://www.tdt.com/support/downloads/ ; and https://www.tdt.com/component/openex-software-suite/). Confocal images were analyzed using the Cell Profiler software (version 3.1.9).
Data analysis	Graph Pad Prism (version 8, La Jolla, CA, USA) or R (version 3.3.3) were used to statistically analyze data sets. Post acquisition analyses of the fiber photometry signals Analyses were performed with Matlab programs (R2017b) based on generic codes from the Lerner and the Gradinaru Labs that can be obtained from https://github.com/talialerner/ and https://github.com/GradinaruLab/.

For manuscripts utilizing custom algorithms or software that are central to the research but not yet described in published literature, software must be made available to editors and reviewers. We strongly encourage code deposition in a community repository (e.g. GitHub). See the Nature Portfolio guidelines for submitting code & software for further information.

Data

Policy information about availability of data

All manuscripts must include a data availability statement. This statement should provide the following information, where applicable:
- Accession codes, unique identifiers, or web links for publicly available datasets
- A description of any restrictions on data availability
- For clinical datasets or third party data, please ensure that the statement adheres to our policy

All the data generated in this study are included within the manuscript's figures, or provided in the supplementary information section and Source Data file. Source Data are provided with this paper. Any additional information are available upon reasonable request.

Field-specific reporting

Please select the one below that is the best fit for your research. If you are not sure, read the appropriate sections before making your selection.

☒ Life sciences ☐ Behavioural & social sciences ☐ Ecological, evolutionary & environmental sciences

For a reference copy of the document with all sections, see nature.com/documents/nr-reporting-summary-flat.pdf

Life sciences study design

All studies must disclose on these points even when the disclosure is negative.

Sample size	All sample size were predetermined based on analyses and results from previous publications (refs. Chaudhury, et al., 2013, Friedman, et al., 2014, Calipari, et al., 2017). In particular, sample size of 5–15 animals were sufficient to determine significance for electrophysiological and fiber photometry recordings and for optogenetic manipulation approaches.
Data exclusions	Only mice with an off-target viral injection were excluded as well as mice with an off-target fiber implantation or damaged optic fiber were removed from this study (n=29/483).
Replication	All attempts at replication were successful.<br>For example, the main behavioral experiments in figure 1 and related supplementary figure 1, were successfully replicated in three independent groups. Further, the main electrophysiological experiments in figure 2 and related supplementary figure 3, were also successfully replicated in independent groups. Similarly, replication of the main effects observed with fiber photometry measurements or induced by optogenetic modulation were successful across independent groups.
Randomization	Animals were randomized by cage prior to surgeries and experimental group assignment. For example, if there were 30 mice in an experiment, with five mice per cage, mice were randomly assigned to be in the socially defeated groups or in the stress naive group in a counterbalanced fashion.
Blinding	Experimenters collecting or analyzing the dataset were blind to the experimental conditions.

Reporting for specific materials, systems and methods

We require information from authors about some types of materials, experimental systems and methods used in many studies. Here, indicate whether each material, system or method listed is relevant to your study. If you are not sure if a list item applies to your research, read the appropriate section before selecting a response.

Materials & experimental systems

n/a	Involved in the study	
☐	☒	Antibodies
☒	☐	Eukaryotic cell lines
☒	☐	Palaeontology and archaeology
☐	☒	Animals and other organisms
☒	☐	Human research participants
☒	☐	Clinical data
☒	☐	Dual use research of concern

Methods

n/a	Involved in the study	
☒	☐	ChIP-seq
☒	☐	Flow cytometry
☒	☐	MRI-based neuroimaging

Antibodies

Antibodies used	The following antibodies were used in the current study:<br>Anti-TH monoclonal antibody from Sigma #T2928 TH-16, lots #110M4777, #088M4846V, #059M4854V; ##105M4874V, #0000091868

	anti-GFP from Invitrogen #A-6455, lots #1853896, #1826342; ##2126798; #2304288; #1826342 and Alexa Fluor 488 and 647 from Jackson Immuno Research, respectively #711-515-152, lots #140422, #143465, ##156009, #143465 and #715-605-150, lot #139301, ##103856, #123283.
Validation	All antibodies for indicated use in our study are fully validated on the manufacturer's website and in our previous publication (Juarez, et al., 2017). Anti-TH monoclonal antibody from Sigma #T2928: https://www.sigmaaldrich.com/content/dam/sigma-aldrich/docs/Sigma/Datasheet/2/t2928dat.pdf Anti-GFP from Invitrogen #A-6455: https://www.thermofisher.com/order/genome-database/dataSheetPdf?producttype=antibody&productsubtype=antibody_primary&productId=A-6455&version=111

Animals and other organisms

Policy information about studies involving animals; ARRIVE guidelines recommended for reporting animal research

Laboratory animals	7-9 week old male experimental mice were used in this study. Heterozygous TH-BAC-Cre mice with a C57BL/6J gene background were bred at Icahn School of Medicine at Mount Sinai. C57BL/6J mice were purchased from Jackson Laboratory and CD1 retired breeder mice aged of 10-16 weeks were purchase from Charles River. Mice were acclimated to the housing facility for 1 week prior to experiments. All mice were group housed and maintained on a 12-h light/dark cycle under stable temperature (22–25°C) and consistent humidity (50±5%) with ad libitum access to food and water. Following CSDS, mice were then singly housed and maintained on a 12-h light/dark cycle with ad libitum access to food and water.
Wild animals	No wild animals were used in the study.
Field-collected samples	No field collected samples were used in the study.
Ethics oversight	All experiments performed are approved by the Institutional Animal Care and Use Committee and comply with institutional guidelines for Animal Care and Use Committee set forth by Icahn School of Medicine at Mount Sinai.

Note that full information on the approval of the study protocol must also be provided in the manuscript.
